# Supplementary material for: MetaRibo-Seq measures translation in microbiomes
Source: Nat Commun. 2020 Jun 29;11:3268. doi: 10.1038/s41467-020-17081-z (PMC7324362; doi:10.1038/s41467-020-17081-z)
Supplement: Supplementary file 10 — Supplementary Data 7 [file 41467_2020_17081_MOESM10_ESM.zip › File2/Confidence_High_Taxonomy/177899_out.krona.html]

Javascript must be enabled to view this page.

members
magnitude
magnitudeUnassigned
count
unassigned
taxon
rank

177899\_out

51

51
superkingdom
2

1239
51
phylum

186801
51
class

186802
51
order

541000
1
family


SRS058070\_contig\_number\_26742
1898205
1
species

50
species
1897049

SRS011084\_contig\_number\_contig-100\_32147.62520SRS011239\_contig\_number\_16426SRS011405\_contig\_number\_contig-100\_11703.81337SRS012849\_contig\_number\_35091SRS014235\_contig\_number\_30310SRS015217\_contig\_number\_23174SRS015431\_contig\_number\_37216SRS015782\_contig\_number\_contig-100\_803.240610SRS016018\_contig\_number\_11685SRS016095\_contig\_number\_27156SRS017821\_contig\_number\_contig-100\_11516.119272SRS019068\_contig\_number\_82036SRS019397\_contig\_number\_2026SRS020233\_contig\_number\_contig-100\_24716.150812SRS020328\_contig\_number\_22435SRS021219\_contig\_number\_contig-100\_20.83117SRS024009\_contig\_number\_contig-100\_7159.68424SRS042628\_contig\_number\_21922SRS043841\_contig\_number\_5513SRS044535\_contig\_number\_7005SRS048981\_contig\_number\_contig-100\_29.54378SRS050998\_contig\_number\_contig-100\_24071.74289SRS051882\_contig\_number\_18669SRS053214\_contig\_number\_contig-100\_19124.61451SRS053649\_contig\_number\_8678SRS055017\_contig\_number\_37299SRS065397\_contig\_number\_contig-100\_27981.27982SRS075341\_contig\_number\_25547SRS075773\_contig\_number\_30498SRS075821\_contig\_number\_13831SRS076929\_contig\_number\_26858SRS077730\_contig\_number\_17665SRS098073\_contig\_number\_contig-100\_10046.10047SRS1041033\_contig\_number\_contig-100\_1581.81656SRS1041039\_contig\_number\_contig-100\_20887.57579SRS1041139\_contig\_number\_contig-100\_2.39101SRS1041143\_contig\_number\_3039SRS143181\_contig\_number\_contig-100\_4710.4710SRS143466\_contig\_number\_19039SRS144297\_contig\_number\_7173SRS144362\_contig\_number\_contig-100\_184.225520SRS146813\_contig\_number\_22739SRS147271\_contig\_number\_contig-100\_14122.160062SRS149325\_contig\_number\_11415SRS893170\_contig\_number\_contig-100\_9997.9998SRS893187\_contig\_number\_14025SRS893292\_contig\_number\_373SRS893295\_contig\_number\_1846SRS893300\_contig\_number\_11458SRS971275\_contig\_number\_38530
